# Surface Functionalization of Citrate-Stabilized Gold Nanoparticles with Various Disease-Specific Nonthiolated Aptamers: RSM-Based Optimization for Multifactorial Disease Biomarker Detection

**DOI:** 10.1021/acssensors.4c02722

**Published:** 2025-02-17

**Authors:** Farbod Ebrahimi, Anjali Kumari, Saqer Al Abdullah, Juan L. Vivero-Escoto, Kristen Dellinger

**Affiliations:** † Department of Nanoengineering, Joint School of Nanoscience and Nanoengineering, North Carolina A&T State University, 2907 East Gate City Boulevard, Greensboro, North Carolina 27401, United States; ‡ Department of Chemistry, University of North Carolina at Charlotte, 9201 University City Boulevard, Charlotte, North Carolina 28223, United States

**Keywords:** biosensor, SERS, bioconjugation, Alzheimer’s disease, diagnosis, surface chemistry, FCCCD

## Abstract

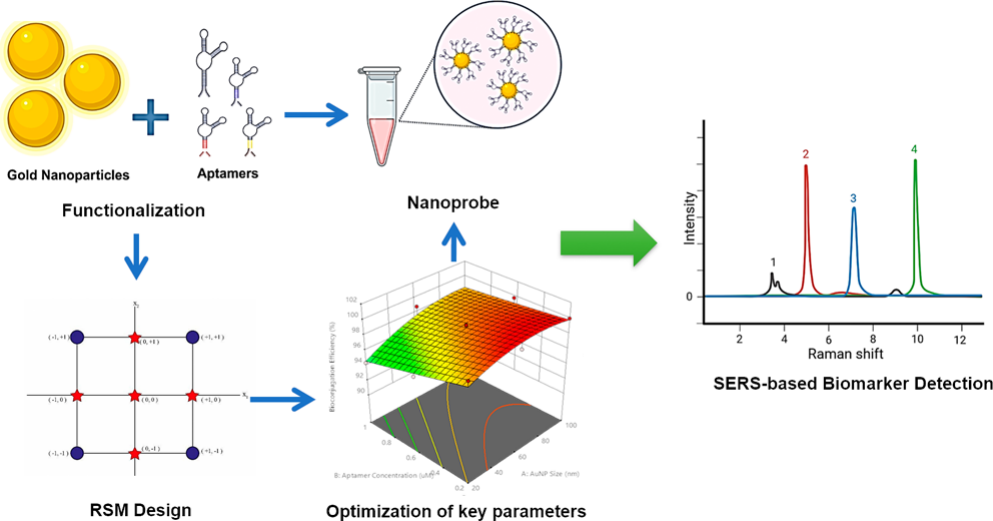

This study focuses on the surface functionalization of citrate-stabilized gold nanoparticles (AuNPs) with disease-specific aptamers to enhance the detection of multifactorial disease (MD) biomarkers. MDs, characterized by complex pathophysiology involving multiple genetic and environmental factors, present significant diagnostic challenges. Aptamers, which are short, single-stranded oligonucleotides with high specificity and affinity for target molecules, have emerged as promising tools for biomarker detection. By utilizing response surface methodology (RSM) and face-centered central composite design (FCCCD), this research systematically optimized the bioconjugation process of AuNPs with different aptamer sequences, focusing on parameters such as AuNP size and aptamer concentration. The developed protocol in this study demonstrated that aptamer-functionalized AuNPs can be optimized for high yield, bioconjugation efficiency, stability, and surface coverage, making them suitable for diagnostic applications, particularly in surface-enhanced Raman spectroscopy (SERS). The findings provide a foundation for the development of customizable nanoprobes that can be adapted for the detection of various biomarkers associated with MDs, potentially improving early diagnosis and therapeutic outcomes.

Despite ongoing advancements in current treatments and diagnostic tools for various multifactorial diseases (MDs), there remains an urgent need for precise and safe therapeutic and diagnostic options. MDs are complex disorders that arise from the interplay of multiple genetic, environmental, and lifestyle factors.^1^ Unlike monogenic diseases, caused by mutations in a single gene,^2^ MDs involve variations in multiple genes, often coupled with external influences such as diet, exposure to toxins, and lifestyle choices.^1,3,4^ Common examples include cardiovascular diseases, diabetes, Alzheimer’s disease (AD), and cancer.^5,6^ These conditions are characterized by their intricate pathophysiology, where numerous biological pathways and mechanisms contribute to disease onset and progression. Understanding and diagnosing MDs pose significant challenges due to their heterogeneity and the cumulative effects of various risk factors.^4^ Detecting abnormal biomarker species is therefore vitally important for understanding MD disease development and progression.^6,7^

Aptamers, derived from the Latin word “aptus” (meaning fit) and the Greek word “meros” (meaning part or unit), are short, synthetic, single-stranded DNA or RNA oligonucleotides capable of folding into unique and complex three-dimensional structures.^8−10^ The flexibility of aptamers allows them to envelop small-molecule targets or fit into gaps on the surfaces of much larger target molecules.^10^ They can bind with high affinity and specificity to a variety of targets, including small molecules, ions, proteins, and even whole living cells, making them valuable for both therapeutic and diagnostic applications.^11,12^ Aptamers offer several attractive features that make them promising targeting agents. Unlike bulky antibodies, aptamers are smaller and have a flexible structure, enabling them to bind to smaller targets or hidden domains that larger antibodies cannot access.^8,10^ Additionally, aptamers can be selected against various targets, including toxic and nonimmunogenic molecules that antibodies cannot recognize.^10,11^ In terms of diagnosis, aptamers have shown significant potential in diagnostic applications due to their ability to bind selectively to biomarkers associated with MDs.^13−15^ Their high specificity and affinity make them excellent candidates for detecting low-abundance targets, which is crucial in early disease diagnosis.^12,16^ For example, based on identifying specific phosphorylation epitopes of tau, DNA aptamers can be developed for their high affinity and selectivity. These aptamers can target tau isoforms and phosphorylation sites with nanomolar sensitivity, making them valuable tools for detecting tau in biofluids, studying tauopathy mechanisms, and potentially serving as therapeutic agents to mitigate AD associated with tau protein aggregation.^9,17^ Aptamer-based sensors (aptasensors) have several advantages over antibody-based biosensors. Aptamers are particularly suitable for MD diagnosis due to their stability, high target affinity, high specificity, and low production costs.^13,18,19^

Gold nanoparticles (AuNPs) have shown promise for biosensing and biomedical applications due to their unique optical properties, facile surface chemistry, and biocompatibility.^6,20^ Their dominant optical property is the excitation of localized surface plasmon resonance (LSPR), which provides selective photon absorption and strong scattering.^21^ This LSPR phenomenon results in the bright color of colloidal AuNPs, which is highly dependent on their composition, size, shape, and distance between them. Moreover, AuNPs have a chemically modifiable surface that allows for their functionalization with recognition molecules such as antibodies and aptamers, making them capable of detecting specific target molecules.^22,23^ In particular, aptamer-functionalized AuNPs have been utilized for the ultrasensitive and specific detection of various biomarkers, making them well suited for selective recognition of disease-associated biomolecular conformations.^24,25^ The combination of (1) the local electromagnetic field enhancement due to LSPR and (2) the chemical enhancement from the adsorption of analyte molecules on the AuNPs-aptamer bioconjugate surface makes this colloidal nanosystem ideal for surface-enhanced Raman spectroscopy (SERS)-based detection.^14,26^

In terms of bioconjugation chemistry of AuNPs and aptamers, thiol-modified aptamers have been widely studied.^27,28^ The Au sites on the surface of AuNPs can be densely occupied through a strong Au–S interaction. Consequently, utilizing thiolated aptamers allows for the easy and controllable loading of ligands onto the AuNPs’ surface.^28,29^ However, thiolated sequences necessitate additional aptamer modification, affecting the final costs and reducing reproducibility due to the easy oxidation of −SH groups.^16^ Another drawback of thiol-modified aptamers is the difficulty in precisely controlling the orientation and conformation of DNA tethered to AuNP surfaces. This is because thiolated DNA molecules interact with the AuNP surface not only through Au–S bonds but also via the amine (AT) groups in nucleotide bases.^16,30,31^ Therefore, there is still a lack of comprehensive studies on aptamer-functionalized AuNPs, particularly concerning alternative aptamer types and modifications, reproducibility, stability, and efficiency.

Considering the advantages of AuNP-aptamer functionality in a wide range of biomedical applications, novel protocols, and optimizations for various types of aptamers are necessary to push the boundaries and further develop nanobioconjugate systems. Therefore, the objective of this study is to optimize the surface functionalization of different-sized citrate-stabilized AuNPs with various disease-specific aptamers, including sequence size as well as modification differences, maximizing coverage and bioconjugation efficiency, specifically for SERS-based applications. To systematically study the effects of key parameters like aptamer concentration and AuNP size on functionalization efficiency, response surface methodology (RSM) with face-centered central composite design (FCCCD) has been utilized. This statistical approach allows for efficient and rigorous optimization of the aptamer-AuNP bioconjugation process for the fabrication of specific and customizable nanoprobes. The optimized aptamer-AuNPs bioconjugate developed through this research will be further translated to the diagnosis of different types of MDs by using SERS.

## Experimental Section

### Reagents and Chemicals

AuNPs with different sizes were purchased from Millipore Sigma. NaCl (NaCl, purity of >99%) was purchased from ThermoFisher. Aptamers (Table S1), T1,^26^ T2,^26^ BT,^19^ and AT,^32^ were purchased from IDT Co, which is specifically used to detect tau. The scrambled aptamer, which has no affinity to tau, was also purchased from IDT Co. Deionized water was used to prepare all of the solutions. Recombinant tau was obtained from Abcam.

### Preparation of Nanobioconjugates

Bioconjugation of AuNPs with manufactured-specified sizes of 20, 50, and 100 nm (citrate-stabilized nanoparticles (NPs), purchased from Millipore Sigma) with different aptamer sequences was performed separately by an *ex situ* bioconjugation procedure. The effects of the aptamer length and chemical modifications with different functional groups on the bioconjugation process and NP performance were investigated. Aptamers of varying lengths, including short (T1) and long (T2) unmodified sequences as well as chemically modified aptamers with biotin (BT) and amine (AT) groups were utilized. These modifications were chosen to assess how different functional groups and sequence lengths influence the binding efficiency, surface coverage, and stability of the AuNP-aptamer conjugates. Instead of the widely used thiol-modified aptamers, biotin- and amine-modified aptamers were chosen to investigate their influence on the bioconjugation process. This approach enabled the evaluation of varying aptamer properties on the performance of the bioconjugation process. Based on the developed and optimized protocol, AuNPs were incubated under salt aging steps, to increase the conjugation efficiency, with the different aptamer sequences to a final concentration of 0.2, 0.6, and 1 μM aptamers, 60 μg/mL of each AuNP size, and 1.5 mM NaCl. The samples were centrifuged based on the AuNP sizes to isolate the AuNP-aptamer bioconjugates and suspended in 1.5 mM NaCl. The final volume of the nanoprobes after centrifugation was 0.5 mL, and the concentration of the pelleted AuNPs was accounted for in the yield calculation. The supernatants were used to measure the unbound ligands by UV–vis. All functionalized samples were stored overnight at 4 °C. Then, the samples were categorized based on AuNP size and type of aptamers for characterization and optimization of the nanoprobe. The characterization of AuNP-aptamer bioconjugates concerning stability (ζ-potential), conjugation efficiency, yield, and surface coverage were carried out using UV–vis spectroscopy (Agilent Cary 7000 UV–vis–NIR) and dynamic light scattering (DLS; Malvern Zetasizer).

### Nanobioconjugate Characterization

After incubation, samples were analyzed by using UV–visible spectroscopy. To separate the incubated AuNPs (product) from any remaining ligands, washing was carried out by centrifugation at different speeds and times with respect to NP size (Table S2). The data was used to calculate yield. Additionally, the absorbance at 260 nm from the supernatant containing unbounded aptamers was used to calculate the conjugation efficiency, as outlined in ref (33).^33^

### Nanobioconjugate Morphology and Size

The morphological studies of samples were carried out using a JSM-IT8000 Schottky JEOL FESEM (JEOL, Japan) and a JEOL JEM-2100 Plus Transmission Electron Microscopy (TEM) (Japan). High-resolution images from scanning electron microscopy (SEM) were captured at 10 keV with a 10 mm working distance using the in-beam secondary electron detector. The outcomes were processed with ImageJ and plotted with Origin software.

### Raman Nanoprobe Preparation

After nanobioconjugation, the 50 nm AuNPs functionalized with BT aptamers were incubated with recombinant tau (1 and 2 nM as the final concentration) for 16 h. The nanoprobes were centrifuged per Table S2 to remove unbound protein and the pellet was resuspended in 0.5 mL of 1.5 mM NaCl. The scrambled aptamer-functionalized 50 nm AuNPs were used as a control nanoprobe. SERS measurements were performed on the nanoprobes prepared in colloidal nanobioconjugates within glass vials. The Raman signal was acquired directly from the AuNP/aptamer/tau colloids. For each sample, Raman measurements were performed in triplicate across three independent samples to ensure accuracy and reproducibility.

### Raman Spectroscopy

Raman spectroscopy measurements were performed by using a portable Raman spectrometer (BWTEK, USA), equipped with a 785 nm laser wavelength. The spectra of the nanoprobes were acquired under the following conditions: an integration time of 1000 ms was set to ensure adequate signal accumulation and a laser power of 50 mW was used to balance between obtaining a strong signal and minimizing potential damage or heating of the samples. The spectrometer’s configuration and settings, including a spectral resolution of 4 cm^–1^, were selected to optimize the resolution and quality of the spectral data for detailed sample analysis.

### Design of RSM Experiments for Gold/Aptamer Bioconjugation

Various parameters such as salt concentration, stability, temperature, incubation time, NP diameter and concentration, initial aptamer concentrations and types, *etc.* influence the bioconjugation process directly or indirectly. However, the study of the effect of each parameter on cumulative bioconjugation at a time is time-consuming and difficult process, particularly when large numbers of input parameters are involved. Hence, keeping view the above fact, in the present work, RSM with three input parameters like NP size, aptamer concentration, and aptamer type is used to study the effects on yield (%), bioconjugation efficiency (%), stability (ζ-potential) (mV), and surface coverage (pmol/cm^2^), while the remaining parameters were fixed at favorable and optimized conditions such as incubation experiments have been performed in fixed and defined conditions for NP initial concentration, time, temperature, and salt concentration. The FCCCD of RSM suggested by Box and Wilson, was used to examine the effect of individual interactions of input parameters on the mentioned responses.^34^ The uncoded levels of three independent input parameters for bioconjugation are given in Table S3. It should be noted that the manufactured 50 nm AuNPs were measured to be larger by DLS. Therefore, for accurate numeric parameters, these particles were assumed to be 60 nm in size. Based on FCCCD recommendations, there are 44 runs comprising various combinations of two numerical variables and one categorical parameter. This parameter includes 12 central points, 16 axial points (points parallel to each variable axis on a circle with a radius of 1.0 and centered at the origin), and 16 factorial points (intersection points of the ±1 coded variable bounds). The combinations of input operating parameters suggested by Design Expert 11 software are detailed in Table S4. Using the bioconjugation experiment results (Table S4), we applied the most suitable model for input-output relationships is applied. Design Expert software (trial version 11, Stat-Ease, USA) was used for RSM regression analysis and optimization of the bioconjugation process with input parameters. The statistical testing of the model was performed by analysis of variance (ANOVA) analysis with *F*-test (*p* < 0.05) to obtain the statistical relationship between input and output parameters.

## Results and Discussion

### UV–Vis Characterization

**Figure 1:**
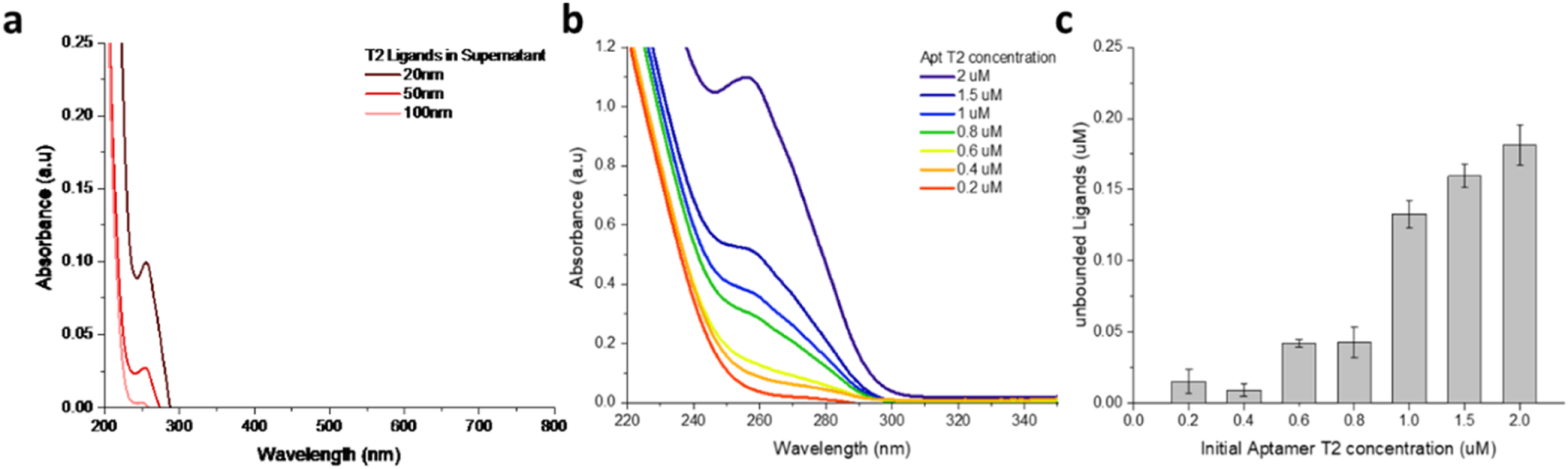
UV–vis analysis showing (a) remaining ligands in the supernatant after centrifugation, (b) absorbance at 260 nm for various aptamer concentrations, and (c) concentration of unbound ligands in the supernatant. Error bars represent the standard deviations from three independent experiments.

To optimize the centrifugation protocol, the UV–vis spectra of supernatants from different-sized AuNPs post-centrifugation were examined (Figure 1a). The lack of peaks corresponding to AuNPs in the supernatants confirms the effectiveness of the optimized centrifuge conditions. From an economic perspective, reducing the costs of nanobioconjugate fabrication is linked to lowering the concentration of applied aptamers. According to Barchanski et al.,^35^ optimal bioconjugate functionality ideally involves a single attached ligand, but in practice, functionality is typically achieved by 1–10 ligands for statistical reasons. However, such a low ligand density may not suffice to stabilize the bioconjugates. Conversely, excessively high ligand concentrations can lead to the development of multilayers on the particle surface. Consequently, two threshold values are defined: the minimum and maximum concentrations of the ligands.

Aptamer T2, characterized by its long unmodified sequence and suitability for physical adsorption were selected for optimization due to its extended tail and physical adsorption properties. Aptamer T2 consists of a long polyadenine (polyA) sequence (Table S1). This polyA region enhances the physical adsorption of the aptamer onto the AuNP surface, as polyA sequences are known for their strong affinity for the Au surfaces. The polyA tail allows for stable and predictable attachment through noncovalent interactions between the adenine bases and Au surface.^16,36^ Additionally, considering that 20 nm AuNPs were the smallest size investigated with the maximum surface area-to-volume ratio (Table S10), this combination was selected to optimize the aptamer concentration. To determine the optimal range of ligand concentration for bioconjugation, AuNPs (20 nm) were conjugated with various concentrations of aptamer T2 (0.2–2 μM).

Figure 1b,**c** illustrates the outcomes of bioconjugation with different ligand concentrations, including the concentration of unbound ligands in the supernatant. Notably, beyond 1 μM, there was a significant increase in unbound ligands in the supernatant, leading to the decision to restrict the range of aptamer concentration between 0.2 and 1 μM. The bioconjugation efficiency and surface coverage are critical parameters in conjugation processes. The surface coverage aptamer is typically controlled during *in vitro* assembly by solution conditions, such as ionic strength, ligand concentration, pH, and diluents. Surface coverage significantly impacts the stability of bioconjugation and the accessibility of bound aptamers for processes like hybridization to complementary strands or enzymatic reactions.^37^ In evaluating the surface coverage of AuNPs/ligands, two methods are commonly used. The first method involves fluorescence-based techniques,^28^ while the second method, utilized in this study, involves measuring the absorbance at 260 nm of the supernatant containing excess ligands.^33^

### SEM and TEM Characterization

**Figure 2:**
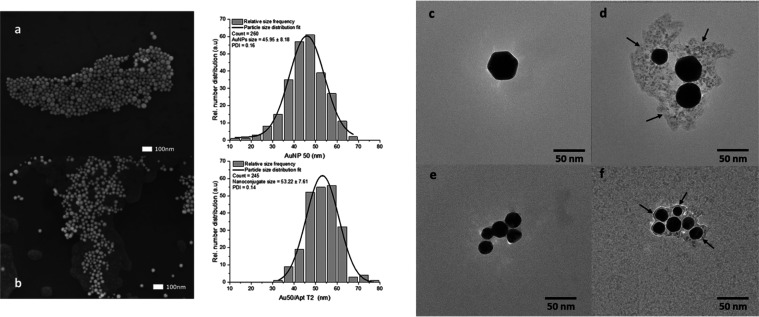
SEM images and particle size distributions for (a) 50 nm AuNPs and (b) 50 nm Au/Aptamer T2 nanoconjugates. TEM images of (c) nonfunctionalized 50 nm AuNPs, (d) 50 nm AuNPs functionalized with Aptamer T2, (e) nonfunctionalized 20 nm AuNPs, and (f) 20 nm AuNPs functionalized with Aptamer T2 (1 μM). The arrows highlight the aptamers surrounding the AuNPs.

Figure 2a shows 50 nm AuNPs before conjugation. These NPs are densely packed together, forming a large cluster. The particles are spherical and seem quite uniform in size. This clustering could be due to the surface properties of the citrate-stabilized NPs in the absence of salt and ligands that can promote aggregation. The SEM image in Figure 2b displays the AuNPs after conjugation with 1 μM aptamer T2. These particles appear more dispersed across the surface with less clustering than that of bare AuNPs. The individual nanoconjugates are still spherical but appear to have a slightly larger average size (by ∼7 nm), validating the bioconjugation process. The reduced clustering might indicate that conjugation with aptamer T2 has altered the surface properties in the presence of salt and ligands, reducing the tendency of the NPs to aggregate.

The histograms (Figure 2a,b) suggest that while the PDI remains almost the same (0.14 and 0.16), indicative of a consistent level of dispersion in both samples, the process of conjugation with aptamer T2 appears to result in a more uniform size distribution of the AuNPs as evidenced by the narrower distribution peak. However, this narrowing could also be attributed to centrifugation, during which smaller NPs may have been removed with the supernatant, resulting in the disappearance of the tail at smaller sizes. This uniformity could be critical for biological applications that require precise particle size; after conjugation, the nanoconjugated 50 nm AuNPs exhibit less clustering and a more uniform and increased size distribution, indicating that the conjugation process impacts the physical properties of the AuNPs.

The TEM images demonstrate the successful functionalization of 50 and 20 nm AuNPs with 1 μM aptamer T2 (Figure 2). The arrows in Figure 2d,f indicate the presence of the aptamer T2 ligands surrounding the AuNPs. The irregularities and surface modifications seen in Figure 2d,f could be due to the long sequence of aptamer T2 attached to the AuNPs. Aptamer T2, being a long sequence, could be folded randomly with the adjacent ligands and does not distribute uniformly across the NP surface. This nonuniform distribution can lead to irregular and asymmetrical shapes after functionalization. Moreover, the functionalization of AuNPs with aptamers, as observed in Figure 2d,f, result in an increase in the overall hydrodynamic diameter of the AuNPs. This size increase is primarily due to the formation of an aptamer layer on the AuNP surface. The presence of these modifications indicates successful conjugation of aptamer T2 to the AuNPs. Additionally, the nonuniform distribution could affect surface coverage, binding sites, and bioconjugation efficiency. As mentioned, Aptamer T2, with its long polyA tail, enhances physical adsorption onto the AuNP surface through noncovalent interactions between the adenine bases and the gold surface. However, this long sequence can result in irregular folding and nonuniform coverage across the NP surface. This irregular distribution can reduce the availability of binding sites for the target molecules and decrease bioconjugation efficiency. This suggests that the length of the aptamer sequence is a crucial factor in bioconjugation. Also, optimizing the concentration of the aptamer during the functionalization process is essential to ensure that the AuNPs are effectively coated without compromising their stability or functionality. This optimization is a vital step in the preparation of aptamer-functionalized AuNPs for their intended applications.

### Effect of Type of Aptamers on Bioconjugation Process

#### Yield

**Figure 3:**
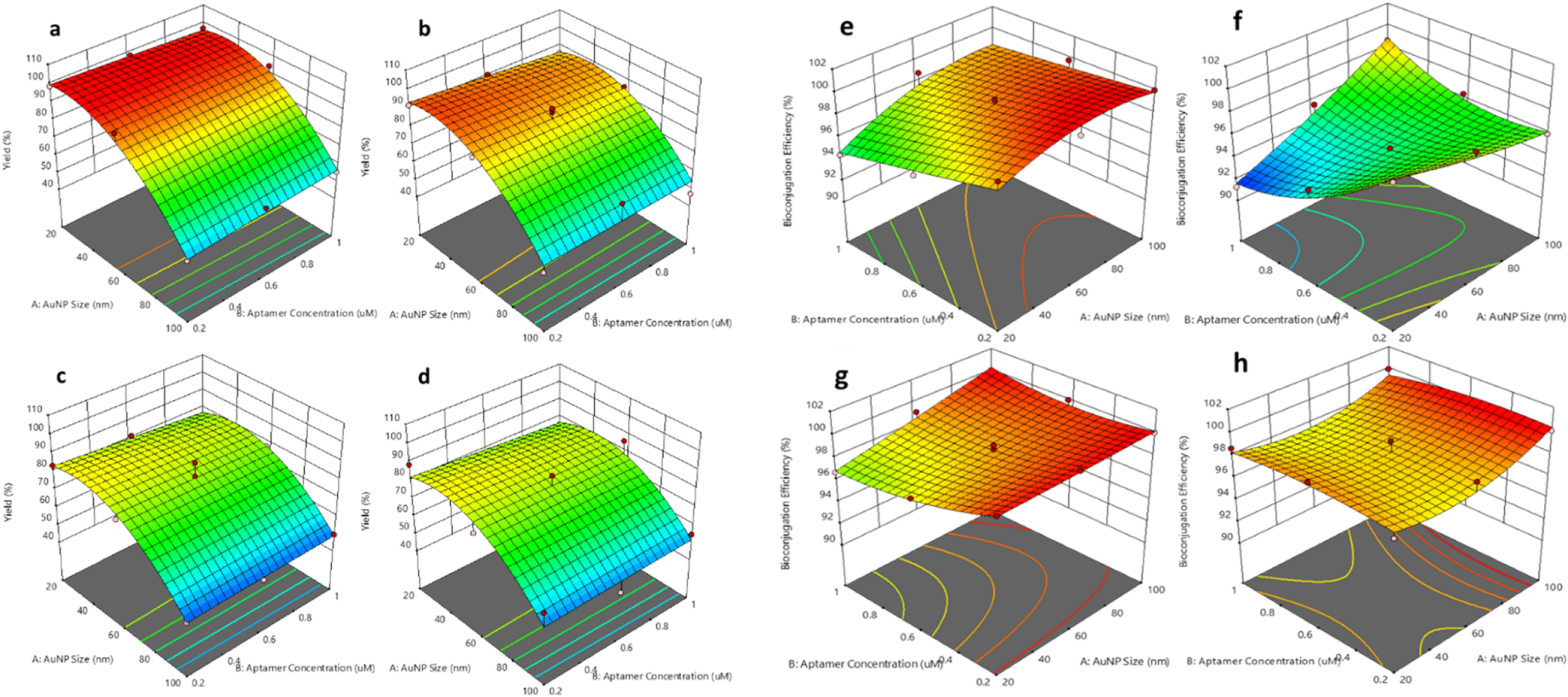
Effect of two-parameter interaction on the yield of bioconjugated aptamers: (a) T1, (b) T2, (c) BT, and (d) AT. Effect of two-parameter interaction on the bioconjugation efficiency of different aptamers: (e) T1, (f) T2, (g) BT, and (h) AT.

Figure 3a shows the highest yield for T1 compared to other aptamers, indicated by the broad area of red. The peak yield is achieved at an intermediate size of AuNPs and at a high T1 aptamer concentration, as suggested by the peak position in the plot.

The yield decreases when either the size of the NPs decreases or the concentration of the T1 aptamer decreases. The yield for T2 also peaks at a higher aptamer concentration (Figure 3b), but the plot suggests that the optimal size of the AuNP may be slightly larger compared to that of T1. The response surface for T2 is less steep compared to T1, indicating that the yield is less sensitive to changes in the two parameters, including AuNP size and aptamer concentration, within the tested range. The yield for the biotin (BT)-modified aptamer shows a different pattern (Figure 3c), with the highest yield occurring at a lower aptamer concentration and a larger AuNP size compared to T1 and T2. The gradient of the surface is more gradual, suggesting that a broader range of conditions can result in a relatively high yield. The pattern for the amine-modified aptamer (AT) is approximately similar to the BT, with a high yield at lower aptamer concentrations (Figure 3d). There is a noticeable plateau of high yield across a range of AuNP sizes, indicating that the amine modification might confer some flexibility in the conditions that yield high bioconjugation efficiency. The yield of the bioconjugation process is influenced by both the size of the AuNPs and the aptamer concentration. However, the exact relationship varies for different aptamer modifications, and the size of sequences peaking at different NP sizes and the chemically modified aptamers (BT and AT) show different sensitivities to the concentration and size variables. The plots indicate that the optimal conditions for the highest yield of bioconjugated NPs are aptamer-specific and depend on the chemical nature of the modification.

#### Nanobioconjugation Efficiency

Citrate stabilization of AuNPs and the use of NaCl for salt aging are important factors in aptamer bioconjugation. Citrate ions bind to the gold surface, imparting a negative charge that can influence the binding affinity and stability of the aptamers. Salt aging with NaCl can affect the aggregation state of the NPs and the binding dynamics of the aptamers. In other words, salt aging with NaCl can lead to charge screening, promoting aptamer adsorption, and potentially inducing NP aggregation if not properly controlled.^29,38^

Comparing the four aptamers, T1 and T2 show the influence of sequence length on bioconjugation efficiency (Figure 3e,f). T1, with a short sequence, requires higher concentrations for peak efficiency, which might indicate a need for dense packing of aptamers on the NP surface. The shorter sequence of T1 might have fewer contact points with the AuNPs, leading to possibly weaker physical adsorption. This might necessitate a higher aptamer concentration to achieve optimal coverage and efficiency. T2, with a longer sequence, likely has a larger surface area that can interact with the AuNPs, potentially allowing for stronger and more numerous van der Waals forces, hydrophobic interactions, or electrostatic interactions, leading to better physical adsorption at lower concentrations. However, in higher concentrations, due to a lack of active sites, the number of bound T2 aptamers has decreased, which leads to a decline in the bioconjugation efficiency.

The modified aptamers (BT and AT) display robust efficiency across a range of conditions (Figure 3g,h). Biotin modification is typically used for a strong affinity to a specific target, such as streptavidin.^15^ The modifications might contribute to a stronger or more stable interaction with AuNPs, allowing for high efficiency even at lower concentrations.^39^ Particularly for AT, the amine groups can partially neutralize the negative charge from the citrate, facilitating a closer interaction with the AuNPs, which can lead to more stable bioconjugates. This might be why the bioconjugation efficiency for AT remains high across a range of conditions. The presence of NaCl could enhance the binding of amine groups by shielding repulsive forces, supporting the broad and high-efficiency region observed for AT. For practical applications, T1 may be suitable for cases where high precision in NP size and aptamer concentration can be achieved. T2 might be preferred when longer sequences are needed for functionality or lower ligand concentrations are desired. BT and AT would be advantageous in scenarios where robustness and stability are critical, such as in variable experimental conditions for biosensors or *in vivo* applications where control over parameters is limited. The choice of aptamer for bioconjugation processes should, therefore, consider not only the efficiency but also the operational conditions, cost implications, and specific applications. The plots provide valuable insights into the behavior of each aptamer type and can guide the optimization of bioconjugation strategies for various biomedical applications.

#### Surface Coverage

**Figure 4:**
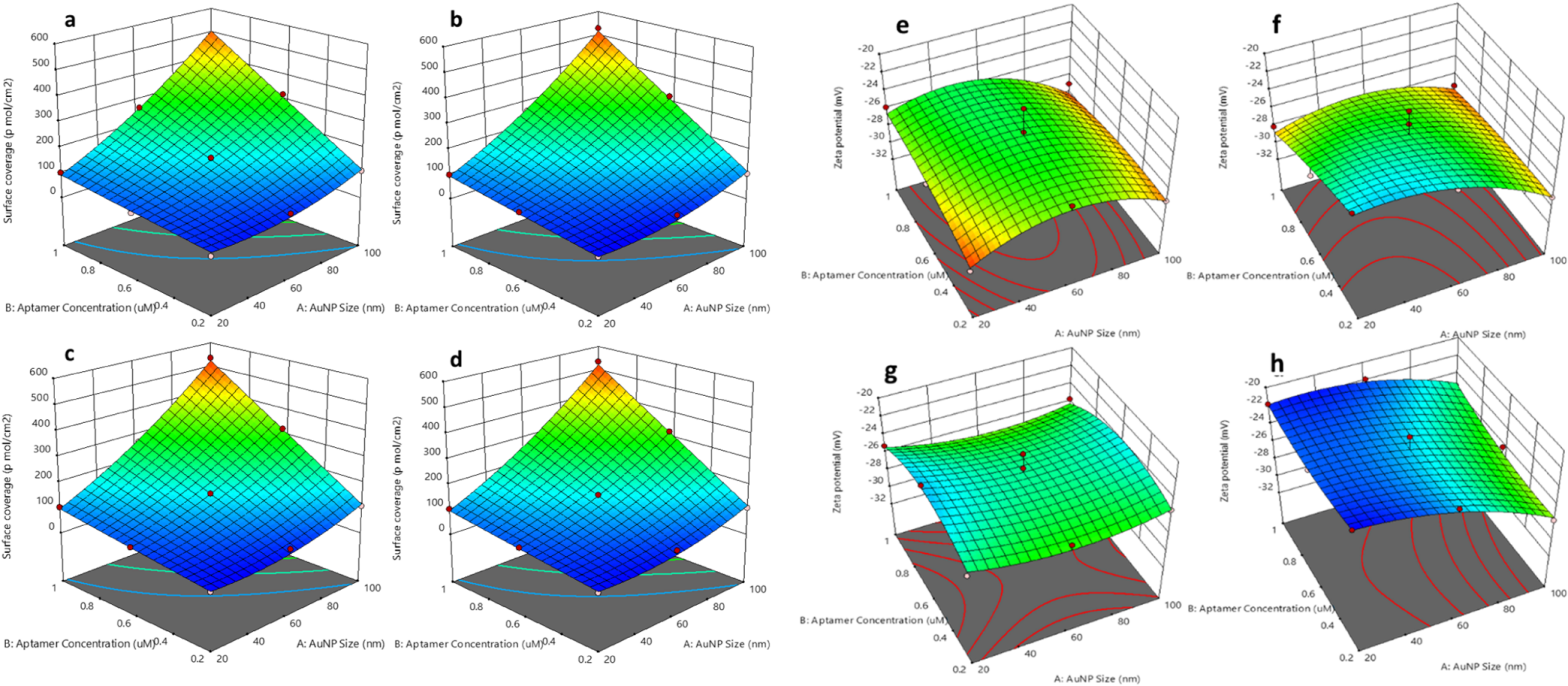
Effect of two-parameter interaction on the surface coverage of different aptamers: (a) T1, (b) T2, (c) BT, and (d) AT. Effect of two-parameter interaction on the stability of different aptamers: (e) T1, (f) T2, (g) BT, and (h) AT.

The T1 plot (Figure 4a) shows a significant increase in surface coverage with an increasing aptamer concentration and NP size, peaking at an intermediate NP size. The shorter sequence likely allows for a higher packing density on the AuNP surface, but this is limited by the physical space available, hence the peak at intermediate sizes. The T2 plot (Figure 4b) indicates a trend similar to that of T1. The BT plot (Figure 4c) exhibits the highest surface coverage among the four aptamers. This suggests that the biotin modification significantly enhances the binding efficiency of the aptamers to the AuNP surface, potentially due to the strong affinity of biotin for the gold surface or the presence of a biotin receptor that promotes denser packing.

Also, the AT plot (Figure 4d) shows a steady and uniform increase in surface coverage with increasing concentration and NP size, which indicates that amine modification provides consistent and stable bioconjugation across a range of conditions. The enhanced surface coverage observed with BT aptamers suggest that biotin modification could be particularly beneficial for applications requiring dense aptamer functionalization of AuNPs. The consistent increase in surface coverage with AT aptamers across all conditions indicates that amine modification may facilitate a more reliable conjugation process, which could be advantageous for applications requiring uniform functionalization.

It is important to note that while surface coverage is critical, the specific application may also require considering other factors such as binding affinity, specificity, and biological activity of the nanobioconjugates. A comparison of surface coverage among the four aptamers suggests that the length of the aptamer and its chemical modifications significantly influence the bioconjugation efficiency. Biotin and amine modifications appear to improve surface coverage compared with unmodified aptamers, potentially offering more effective strategies for AuNP functionalization in biomedical applications.

#### Stability

To study colloid stability, we must apply the Derjaguin–Landau–Verwey–Overbeek (DLVO) theory. The DLVO theory examines the equilibrium between electrostatic repulsion and van der Waals attraction forces.^40^ Particles that are electrostatically stabilized depend on factors such as the concentration of electrolytes (1.5 mM NaCl in our work), the pH of the solution (6.7 ± 0.2), the electrical potential on the particle surface, and the Hamaker constant (*A*), which characterizes van der Waals particle–particle interactions. These parameters, as outlined by the DLVO theory, influence the surface charges by affecting the thickness of the electrical double layer and ultimately lead to particle agglomeration or stability.^40,41^ Regarding the chemistry of aptamer bioconjugation, although the mechanisms behind the adsorption of aptamers onto AuNPs remains unclear and several potential explanations have been proposed:^42^ (1) van der Waals forces attracting the bases of the aptamer to the AuNPs, without replacing the citrate molecules;^43^ (2) hydrophobic interactions between hydrophobic residues of the DNA and highly hydrated anions;^44^ (3) conditions such as high salt concentration, low pH, or the presence of ethanol reduce electrostatic repulsion and facilitate the aptamer’s approach to the Au surface, resulting in the displacement of citrate through noncovalent interactions.^45^ Furthermore, (4) other studies suggest that a direct binding between the cytosine and adenine bases of the aptamer and the citrate on the surface of the AuNPs may also be possible.^46,47^

For aptamer T1 (Figure 4e), as AuNP size increases from 20 to 100 nm, the ζ-potential becomes more negative. This can be attributed to the less curved surface of bigger NPs,^29^ which allows for more binding of the negatively charged citrate ions, enhancing the electrostatic stabilization. Higher aptamer concentrations also result in more negative ζ-potential values. This could be due to the increased number of aptamers providing additional negative charges, enhancing repulsion between particles and, thus, stability. The flexible and smaller structure of T1 aptamers might allow them to bind efficiently to the surface, increasing the packing density. This closer packing enhances the surface charge by allowing more aptamer molecules to interact with the AuNP surface, facilitated by van der Waals forces and possibly direct binding between the cytosine and adenine bases (polyA region) of the aptamer and the citrate on the AuNP surface. Aptamer T2 (Figure 4f), being a longer unmodified sequence, shows a similar trend to T1 with increasing AuNP size leading to more negative ζ-potentials. The longer sequences might have more binding sites, allowing them to interact more with the NP surface. At higher aptamer concentrations, the negative ξ-potential values suggest improved stability. This could be because the longer sequences can cover more surface area on the NPs, providing more uniform coverage and better stabilization through enhanced electrostatic repulsion, van der Waals forces, and hydrophobic interactions.

For the biotin-modified aptamer (BT) (Figure 4g), the ζ-potential decreases with increasing AuNP size, but the effect of the aptamer concentration is less pronounced. The biotin modification likely enhances the stability of the NP-aptamer conjugate due to the strong affinity between biotin and the gold surface. This strong binding might reduce the dependence on aptamer concentration for stability, as biotin provides a robust anchor point, resulting in good stability even at lower concentrations. The biotin–gold interaction ensures a stable conjugate, which can explain why ζ-potential values remain low across varying concentrations. The amine-modified aptamer (AT) shows a significant decrease in the ζ-potential with increasing AuNP size, especially at lower aptamer concentrations (Figure 4h). The amine groups can interact strongly with the gold surface, providing good stabilization through a combination of electrostatic and covalent interactions. At higher AuNP sizes, the decreased particle curvature allows for more extensive binding of these amine groups, leading to better stabilization. The less drastic change in ζ-potential with higher aptamer concentrations might be due to the already strong binding affinity of the amine groups, making additional aptamer concentration less impactful on stability.

### Optimization of Nanobioconjugation Parameter for Various Aptamers

A critical aspect of this study was to determine the optimal conditions for bioconjugation (for maximum efficiency and surface coverage) by minimizing the aptamer concentrations regarding different aptamer types. Results indicated that the initial concentration (∼0.5 μM) was optimal for all aptamers with a selected AuNP size. Using the numerical optimization method in Design Expert 11, the results (Table 1) demonstrated that T1 had the highest bioconjugation efficiency (99.47%) and yield (80.73%), making it an optimal choice for applications requiring both high efficiency and yield. T2, while showing a slightly lower bioconjugation efficiency (95.30%), provided good surface coverage (177.666 pmol/cm^2^) and a yield of 75.27%, making it suitable for applications that benefit from longer sequences. The biotin-modified aptamer (BT) exhibited high bioconjugation efficiency (99.01%) and surface coverage (180.44 pmol/cm^2^) but had a lower yield (71.46%), suggesting that its strong affinity for gold surfaces is advantageous for dense functionalization applications. The amine-modified aptamer (AT) achieved the highest surface coverage (198.28 pmol/cm^2^) and high bioconjugation efficiency (98.90%), indicating its suitability for stable and robust applications despite a lower yield (70.08%).

**Table 1:** Numerical Optimized Parameter for Minimize Initial Aptamer Concentration

categoric parameter	optimized numeric parameter value		expected outcome		
aptamer type	AuNP size (nm)	initial aptamer concentration (μM)	bioconjugation efficiency (%)	surface coverage (pmol/cm^2^)	yield (%)
T1	74.92	0.51	99.47	173.481	80.73
T2	78.97	0.49	95.30	177.666	75.27
BT	74.28	0.52	99.01	180.44	71.46
AT	78.42	0.53	98.90	198.28	70.08

The optimized AuNP sizes for each aptamer type provide valuable insights into how NP size influences bioconjugation efficiency, surface coverage, and yield. For aptamer T1, which is an unmodified short sequence, the optimal AuNP size is approximately 74.92 nm. This moderately small size likely offers a sufficient surface area for effective binding. For aptamer T2, an unmodified long sequence, optimal performance is achieved with slightly larger AuNPs of around 78.97 nm. The larger NP size may provide more surface area to accommodate the longer sequence of T2. For the BT aptamer, the optimal AuNP size is approximately 74.28 nm, similar to that of T1. This suggests that biotin modification does not require significantly larger nanoparticles for effective binding. The AT aptamer performs best with AuNPs of around 78.42 nm. This Au size supports the robust binding characteristics of amine modification. According to the results for the optimum size of NPs, between 50 and 80 nm might be the best option for the nanoprobe fabrication process regarding SERS biosensing. These results underscore the importance of selecting aptamer types based on the application’s specific needs, balancing factors such as bioconjugation efficiency, surface coverage, and yield to achieve the desired outcomes.

### SERS Nanoprobe Detection

The nanoprobes were synthesized based on optimized value, including 50 nm AuNPs and 0.5 μM concentration of BT. Raman spectroscopy was employed to analyze the interaction between the nanoprobes and the tau protein.^48^ The studies show that the Raman peaks of tau protein can shift slightly depending on the buffer solution used.^48^ The SERS measurements were performed in triplicate using three independent batches of samples to ensure reproducibility.

**Figure 5:**
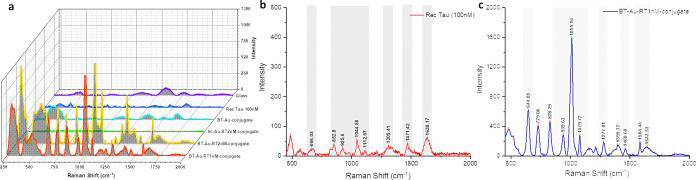
SERS-based detection of recombinant tau protein: (a) Raman spectra of various colloidal samples, including empty glass (purple), recombinant tau protein (100 μM, blue), biotin-modified aptamer/AuNP conjugate (cyan), scrambled aptamer-functionalized 50 nm AuNPs (green), biotin-modified aptamer/AuNP-recombinant tau (2 nM, yellow), and biotin-modified aptamer/AuNP-recombinant tau (1 nM, red). (b) Raman spectrum of recombinant tau protein. (c) SERS spectrum of the developed nanoprobe (biotin-modified aptamer/AuNP-recombinant tau 1 nM).

Figure 5b,c depicts the Raman spectra of the pure recombinant tau protein and the developed SERS nanoprobe. The spectra show distinct peaks corresponding to the amide I (1638.17 and 1643.33 cm^–1^), amide II (1471.42 and 1460.68 cm^–1^), and amide III (1309.41 and 1277.81 cm^–1^) bands for pure recombinant tau (Figure 5b) and nanoprobe (Figure 5c), respectively. These peaks were assigned in the spectrum with the amide I band primarily attributed to C=O stretching, the amide II band to the out-of-phase combination of N–H in-plane bending and C–N stretching, and the amide III band to the in-phase combination of N–H in-plane bending and C–N stretching. Additionally, the peak at 1399 cm^–1^ in Figure 5c is due to Cα–H bending. The aromatic modes of tyrosine and phenylalanine are centered around 666.03 and 644.85 cm^–1^. The peak at 729.06 cm^–1^ is due to C–S stretching, and the skeletal region, which consists of C–C and C–N stretching from 850 to 1150 cm^–1^, was also detected.^48,49^ The results indicate that the SERS spectra of the tau protein bound to the AuNPs show significant shifts and intensity changes compared to those of the pure tau protein. These changes are indicative of successful bioconjugation and interactions between the tau protein and the functionalized AuNPs. The shifts in the Raman peaks, particularly in the amide I, II, and III bands, suggest alterations in the protein’s secondary structure upon binding to the nanoprobes. This binding is further confirmed by the presence of characteristic peaks corresponding to C=O stretching, N–H in-plane bending, C–N stretching, Cα–H bending, C–S stretching, and aromatic modes of tyrosine and phenylalanine.

The optimized synthesis of AuNPs functionalized with BT aptamers, followed by incubation with recombinant tau, resulted in increased protein capture by the nanoprobes, leading to significant enhancement in direct SERS sensing (Figure 5a), confirming successful fabrication and providing insights into the molecular interactions between the nanoprobes and tau. This study demonstrates the potential of developed bioconjugates for SERS-based detection and analyzing protein interactions at the nanoscale, contributing to the development of advanced diagnostic tools for AD.

## Conclusions and Outlook

This study systematically optimized the surface functionalization of citrate-stabilized AuNPs with various disease-specific aptamers using the RSM with an FCCCD for biomedical applications. Conditions that maximize surface coverage and bioconjugation efficiency were identified by carefully examining key parameters such as the aptamer concentration, AuNP size, and aptamer sequence type. The findings revealed that optimal bioconjugation conditions vary significantly depending on the aptamer type, underscoring the need for specific optimization for each aptamer. The study also demonstrated that the bioconjugation efficiency and stability of AuNP-aptamer complexes are strongly influenced by the sequence length and chemical modifications of the aptamers. Modified aptamers, such as biotin- and amine-modified sequences exhibited enhanced stability and a higher bioconjugation efficiency across a range of conditions. Through our developed bioconjugation protocol, we achieved a relatively high bioconjugation efficiency for all aptamer types. Notably, Aptamer T1 emerged as the most efficient with a bioconjugation efficiency of 99.47% and a yield of 80.73% at a low initial concentration of 0.51 μM and an AuNP size of 74.92 nm. While Aptamer T2 provided slightly less efficiency (95.30%), it offered higher surface coverage (177.666 pmol/cm^2^) but with a lower yield (75.27%). Aptamers BT and AT achieved the highest surface coverage (180.44 and 198.28 pmol/cm^2^, respectively) but required higher initial aptamer concentrations and resulted in lower yields. The results suggest that AuNP sizes between 50 and 80 nm is optimal for nanoprobe fabrication in SERS biosensing applications. These findings emphasize the importance of selecting aptamer types based on specific biomedical application needs and balancing factors such as bioconjugation efficiency, surface coverage, and yield. Furthermore, the successful customizable nanoprobe fabrication, as confirmed by SERS results, provide insights into molecular interactions between the nanoprobes and tau and significant enhancement in signal intensity, highlighting the potential of the developed AuNP/Aptamer bioconjugates for SERS-based detection and analysis of protein interactions at the nanoscale, contributing to the advancement of diagnostic tools for MD, such as AD.

## Supporting Information Available

Additional experimental details, calibration lines, ANOVA results, and optimization parameters for aptamer-conjugated AuNPs; aptamer specifications, centrifuge conditions for different AuNP sizes, RSM design parameters, FCCCD matrix response values, and ANOVA modeling results; UV–vis calibration lines and optimization parameters for aptamer sequences T1, T2, BT, and AT; ligand and particle concentrations and numerical optimization graphs (PDF)

## Abbreviations

AuNPs: gold nanoparticles
MDs: multifactorial diseases
AD: Alzheimer’s diseases
RSM: response surface methodology
FCCCD: face-centered central composite design
LSPR: localized surface plasmon resonance
SERS: surface-enhanced Raman spectroscopy
