# Supplementary material for: Surface Functionalization of Citrate-Stabilized Gold Nanoparticles with Various Disease-Specific Nonthiolated Aptamers: RSM-Based Optimization for Multifactorial Disease Biomarker Detection
Source: ACS Sens. 2025 Feb 17;10(2):944–53. doi: 10.1021/acssensors.4c02722 (PMC11877523; doi:10.1021/acssensors.4c02722)
Supplement: Supplementary file 1 — se4c02722_si_001.pdf [file se4c02722_si_001.pdf]

Supplementary Information

Surface Functionalization of Citrate-Stabilized Gold Nanoparticles with Various Disease-Specific Non-Thiolated Aptamers: RSM-Based Optimization for Multifactorial Disease Biomarker Detection

Farbod Ebrahimi[1], *Anjali Kumari*[1], *Saqer Al Abdullah*[1], *Juan L. Vivero-Escoto*[2], *Kristen Dellinger*[1,*]

[1]Department of Nanoengineering, Joint School of Nanoscience and Nanoengineering, North Carolina A&T State University, 2907 E Gate City Blvd, Greensboro NC 27401, USA

[2]Department of Chemistry, University of North Carolina at Charlotte, 9201 University City Boulevard, Charlotte NC 28223, USA

*E-mail: kdellinger@ncat.edu

Contents

Tables S1 to S10

Figures S1 to S3

Aptamer specifications

Table S1. *Aptamer specifications*

Aptamer Name	Sequence	Number of amino acids	Specificity for work
**T1**[1]	5′-AAAAATTTTTGCGGAGCGTGGCAGG-3′	25	Short unmodified sequence
**T2**[1]	5′-AAAAAAAAAAAAAAAAAAAAAAAAAAAAAAATTTTTGCGGAGCGTGGCAGG-3′	50	Long unmodified sequence
**BT**[2]	5′-Biotin-GCGGAGCGTGGCAGG-3′	15	Biotin modified sequence
**AT**[3]	5′-H_2N-GCGGAGCGTGGCAGG-3′	15	Amine modified sequence

Centrifuge conditions

Table S2. *Centrifuge conditions for different AuNPs sizes*

AuNPs size (nm)	Speed (g)	Time (min)
20	15000	30
50	2000	15
100	800	10

RSM design levels, parameters and responses

RSM is a statistical and mathematical technique that is used for modeling and analysis of response/output variable which is influenced by several input parameters[4]. The purpose of RSM is to simultaneously optimize the levels of selected three input parameters, including two numeric factors and one categorical factor, to obtain the maximum responses regarding the interest output factors[4]. Table S3 shows the minimal ($X_{i,\text{ min}}$), the mid-range ($X_{i,\text{ mid}}$) and the maximal ($X_{i,\text{ max}}$) levels for two numeric parameters, which corresponds to −1, 0 and +1 levels, respectively, in terms of coded variable X_i defined by Eq. (1).

$$X_i = \frac{2\,(X_{actual} - \overline{X})}{X_{i,max} - X_{i,min}} \qquad (1)$$

Where, $\overline{X} = \; = \frac{X_{i,max}+X_{i,min}}{2}$

Table S3. *Coded and real levels of input parameters involved in experimental design*

**Numeric Factors**	Name	Low level $X_{i,min}$= -1	Mid-level $X_{i,mid}$= 0		High level $X_{i,max}$= 1
**X_1**	AuNPs size	20	60		100
**X_2**	Aptamer concentration	0.2	0.6		1
**Categorical Factor**		Level 1	Level 2	Level 3	Level 4
**X_3**	Aptamer sequence type	T1	T2	BT	AT

Table S4. *FCCCD matrix and the response values for the AuNPs-aptamer bio-conjugates*

No.	Type	X_1:AuNP Size (nm)	X_2:Aptamer Concentration (uM)	X_3:Aptamer type	Yield(%)	Bioconjugation Efficiency(%)	Surface coverage (p mol/cm^{-2})	ζ-potential (mV)
**1**	axial	20	0.2	AT	88.6348	97.8659	20.7724	-21.56
**2**	axial	100	0.2	AT	57.4509	99.9903	107.15	-27.71
**3**	axial	20	1	AT	75.3911	98.6587	102.567	-21.78
**4**	axial	100	1	AT	54.5392	99.9323	536.059	-25.3
**5**	fact	20	0.6	AT	77.9271	98.962	64.3089	-22.41
**6**	fact	100	0.6	AT	44.0194	99.9903	321.636	-26.2
**7**	fact	60	0.2	AT	74.4518	98.9226	53.5696	-22.87
**8**	fact	60	1	AT	85.5352	96.7089	257.357	-21.86
**9**	center	60	0.6	AT	74.6396	97.4601	153.532	-24.47
**10**	center	60	0.6	AT	84.8777	99.1971	159.542	-22.03
**11**	center	60	0.6	AT	71.8218	98.1791	154.688	-24.19
**12**	axial	20	0.2	BT	83.4688	99.8493	21.4121	-25.3
**13**	axial	100	0.2	BT	47.4309	99.971	107.191	-25.22
**14**	axial	20	1	BT	74.5401	96.7361	102.65	-25.18

**15**	axial	100	1	BT	49.4671	99.9826	536.018	-25.63
**16**	fact	20	0.6	BT	81.3085	97.7996	60.3444	-22.9
**17**	fact	100	0.6	BT	46.3675	99.9839	321.615	-24.25
**18**	fact	60	0.2	BT	77.0761	99.9998	53.611	-25.62
**19**	fact	60	1	BT	77.5514	98.9865	268.019	-27.54
**20**	center	60	0.6	BT	87.132	98.63	145.763	-22.69
**21**	center	60	0.6	BT	76.2364	98.976	157.019	-26.9
**22**	center	60	0.6	BT	79.7117	97.6287	154.361	-24.25
**23**	axial	20	0.2	T1	98.4032	99.171	21.4382	-30.06
**24**	axial	100	0.2	T1	52.0032	99.9807	107.201	-29.37
**25**	axial	20	1	T1	96.7125	94.4165	100.163	-25.8
**26**	axial	100	1	T1	55.2906	97.7023	486.265	-29.02
**27**	fact	20	0.6	T1	97.9336	96.086	56.6687	-27.75
**28**	fact	100	0.6	T1	56.9813	99.7359	320.817	-30.22
**29**	fact	60	0.2	T1	94.9279	99.3806	53.6007	-26.25
**30**	fact	60	1	T1	93.8947	98.7017	265.38	-27.43
**31**	center	60	0.6	T1	88.6348	99.2229	158.495	-25.16
**32**	center	60	0.6	T1	87.6955	98.5458	158.915	-22.56
**33**	center	60	0.6	T1	89.5798	98.807	159.584	-27.44
**34**	axial	20	0.2	T2	91.5465	98.9903	21.4424	-23.27
**35**	axial	100	0.2	T2	50.1246	95.941	101.798	-28.91
**36**	axial	20	1	T2	85.6291	91.2594	97.8504	-28.09
**37**	axial	100	1	T2	46.9311	98.5773	529.556	-29.29
**38**	fact	20	0.6	T2	90.3255	94.6839	64.323	-26.76
**39**	fact	100	0.6	T2	63.9319	96.4355	321.459	-29.31
**40**	fact	60	0.2	T2	86.0048	97.8586	52.9992	-24.42
**41**	fact	60	1	T2	85.5352	95.5314	261.438	-28.34
**42**	center	60	0.6	T2	84.2202	94.0646	141.594	-24.28
**43**	center	60	0.6	T2	90.0437	93.9377	149.196	-22.85
**44**	center	60	0.6	T2	92.1101	94.7501	143.383	-27.72

The detailed modeling results and ANOVA for RSM variables

Yield

The yield was calculated based on experimental data from UV-vis measurements of AuNP products and initial concentration, by given Eq. (2);

$$Yield\ (\%) = \frac{AuNP\ concentration\ of\ product_{@\ related\ SPR\ peak}}{Initial\ AuNP\ concentration_{@\ related\ SPR\ peak}} \times 100 \qquad (2)$$

The concentration of AuNPs was determined using UV-vis-derived calibration lines (see **Figure S1**). Commonly used models, including linear, two-factor interaction (2FI), reduced cubic, and quadratic, were tested and analyzed using ANOVA to identify the best correlation between yield and input parameters for various aptamers. The quadratic model was found to fit the experimental data significantly better than the other models, and thus, only the quadratic model is discussed

here. Through regression analysis of the experimental yield data, the equation for the quadratic model was developed and is presented in coded factors in Eq. (3).

$$Yield\,(\%) = 84.12 - 17.39X_1 - 0.8544X_2 - 4.34X_{3,1} - 5.16X_{3,0} + 6.82X_{3,-1} + 1.81X_1X_2 + 3.06X_1X_{3,1} + 1.38X_1X_{3,0} - 4.08X_1X_{3,-1} + 0.0091X_2X_{3,1} - 0.2151X_2X_{3,0} + 0.9484X_2X_{3,-1} - 14.63X_1^2 - 0.1023X_2^2 \quad (3)$$

The equation in terms of coded factors can be used to predict the response for given levels of each factor. By default, high levels of the factors are coded as +1, and low levels are coded as -1. This coded equation is useful for identifying the relative impact of the factors by comparing their coefficients. The significance of the developed quadratic model is determined by ANOVA analysis. The ANOVA analysis followed by F-test is applied to estimate the significance of each term. The large F-value shows that most of the variation in the output can be explained by the developed regression equation. The associated p-value is also used to estimate, whether F is large enough to indicate statistical significance.

Table S5. *ANOVA for RSM variables fitted to Yield quadratic model*

Source	Sum of Squares	df	Mean Square	F-value	p-value	Significance
**Model**	10934.68	14	781.05	31.19	< 0.0001	significant
**X_1-AuNP Size**	7255.21	1	7255.21	289.77	< 0.0001	
**X_2-Aptamer Concentration**	17.52	1	17.52	0.6998	0.4097	
**X_3-Aptamer type**	1090.04	3	363.35	14.51	< 0.0001	
**X_1X_2**	52.56	1	52.56	2.10	0.1581	
**X_1X_3**	168.15	3	56.05	2.24	0.1049	
**X_2X_3**	8.98	3	2.99	0.1196	0.9479	
**X_1^2**	2167.46	1	2167.46	86.57	< 0.0001	
**X_2^2**	0.1061	1	0.1061	0.0042	0.9485	
**Residual**	726.10	29	25.04			
**Lack of Fit**	534.50	21	25.45	1.06	0.4951	insignificant
**Pure Error**	191.61	8	23.95			
**Cor Total**	11660.79	43				

The results of ANOVA for quadratic model are shown in **Table S5** and it is perceptible that the individual, second order and interaction terms. The p-value of quadratic model is < 0.0001 which clearly confirm excellent fit of experimental data. The value of correlation coefficient, Rsquare, gives the idea about accuracy of the suggested model. The value of R^2 gives the proportion of total variation in the yield predicted by developed model and 0.9377 for quadratic model ensures a proper fit.

Nanobioconjugation efficiency

The bio-conjugation efficiency was calculated based on experimental data from UV-vis measurements of initial concentration of ligands (C_0) and concentration of unbounded ligand (C_1) at 260 nm, by given Eq. (4);

$$Bioconjugation\ (\%) = \frac{C_0 - C_1}{C_0} \times 100 \quad (4)$$

The concentration of ligands was identified based on UV-vis-derived calibration lines (see **Figure S2**). For bio-conjugation efficiency, it has been observed that the reduced cubic model fits the experimental data significantly well in comparison to other models. Through regression analysis of the experimental bio-conjugation efficiency data, the specific equation for the reduced cubic model is derived and provided in coded factors Eq. (5);

$$Bioconjugation\ (\%) = 97.60 + 0.9893X_1 - 0.8636X_2 + 0.6593X_{3,1} + 0.9893X_{3,0} + 1.32X_{3,-1} + 0.9448X_1X_2 - 0.2516X_1X_{3,1} - 0.0639X_1X_{3,0} + 0.3015X_1X_{3,-1} + 0.6172X_2X_{3,1} + 0.1778X_2X_{3,0} - 0.4217X_2X_{3,-1} + 0.1376X_1^2 + 0.4393X_2^2 - 1.16X_1X_2X_{3,1} - 0.1636X_1X_2X_{3,0} - 0.3258X_1X_2X_{3,-1} + 1.11X_1^2X_{3,1} - 0.0988X_1^2X_{3,0} - 1.23X_1^2X_{3,-1} - 0.8504X_2^2X_{3,1} + 0.2010X_2^2X_{3,0} - 0.4019X_2^2X_{3,1} \quad (5)$$

Table S6 displays the ANOVA results for the reduced cubic model, indicating significant contributions from individual, second- and third order, and interaction terms. The p-value for the reduced cubic model (< 0.0001) confirms its excellent fit to the experimental data. The correlation coefficient, Rsquare, reflects the accuracy of the model, with an R^2 value of 0.9353 for the reduced cubic model ensuring a satisfactory fit by explaining a large proportion of the total variation in the predicted bio-conjugation efficiency.

Table S6. *ANOVA for RSM variables fitted to Bioconjugation Efficiency Reduced Cubic model*

Source	Sum of Squares	df	Mean Square	F-value	p-value	Significance
**Model**	171.25	23	7.45	12.57	< 0.0001	significant
**X_1-AuNP Size**	23.49	1	23.49	39.67	< 0.0001	
**X_2-Aptamer Concentration**	17.90	1	17.90	30.23	< 0.0001	
**X_3-Aptamer type**	78.00	3	26.00	43.90	< 0.0001	
**X_1X_2**	14.28	1	14.28	24.12	< 0.0001	
**X_1X_3**	0.9512	3	0.3171	0.5354	0.6634	
**X_2X_3**	4.38	3	1.46	2.46	0.0920	
**X_1^2**	0.1920	1	0.1920	0.3242	0.5755	
**X_2^2**	1.96	1	1.96	3.30	0.0842	
**$X_1X_2X_3$**	16.74	3	5.58	9.42	0.0004	
**$X_1^2X_3$**	7.11	3	2.37	4.00	0.0220	
**$X_2^2X_3$**	5.14	3	1.71	2.89	0.0606	
**Residual**	11.84	20	0.5922			
**Lack of Fit**	8.73	12	0.7272	1.87	0.1913	insignificant
**Pure Error**	3.12	8	0.3898			
**Cor Total**	183.09	43				

Surface coverage

In terms of surface coverage, the quadratic model demonstrates a superior fit to the experimental data compared to other models. Through regression analysis of the experimental surface coverage

data, the specific equation for the quadratic model is developed and provided in coded factors Eq. (6);

$$Surface\ coverage\ (pmole/cm^{-2}) = 154.94+127.62X_1+117.55X_2+2.09X_{3,1}+1.80\ X_{3,0}-1.79X_{3,-1}+84.15X_1X_2+1.91X_1X_{3,1}+2.45X_1X_{3,0}-4.95X_1X_{3,-1}+1.53X_2X_{3,1}+3.20X_2X_{3,0}-5.95X_2X_{3,-1}+33.57X_1^2+0.4165X_2^2 \quad (6)$$

The ANOVA results for the quadratic model, as presented in **Table S7**, indicate significant contributions from individual, second-order, and interaction terms. With a p-value of < 0.0001, the quadratic model confirms an excellent fit to the experimental data. The correlation coefficient, Rsquare, provides insight into the accuracy of the model. A high R^2 value of 0.9954 for the quadratic model ensures that an exceptional proportion of the total variation in the predicted surface coverage is accounted for, indicating an excellent fit.

Table S7. *ANOVA for RSM variables fitted to Surface coverage Quadratic model*

Source	Sum of Squares	df	Mean Square	F-value	p-value	Significance
**Model**	8.488E+05	14	60630.86	451.84	< 0.0001	significant
**X_1-AuNP Size**	3.909E+05	1	3.909E+05	2912.87	< 0.0001	
**X_2-Aptamer Concentration**	3.316E+05	1	3.316E+05	2471.30	< 0.0001	
**X_3-Aptamer type**	167.62	3	55.87	0.4164	0.7425	
**X_1X_2**	1.133E+05	1	1.133E+05	844.33	< 0.0001	
**X_1X_3**	207.04	3	69.01	0.5143	0.6757	
**X_2X_3**	297.05	3	99.02	0.7379	0.5380	
**X_1^2**	11417.00	1	11417.00	85.08	< 0.0001	
**X_2^2**	1.76	1	1.76	0.0131	0.9097	
**Residual**	3891.43	29	134.19			
**Lack of Fit**	3769.66	21	179.51	11.79	0.0006	significant
**Pure Error**	121.77	8	15.22			
**Cor Total**	8.527E+05	43				

Stability

Regarding stability (ζ-potential), it has been noted that the reduced cubic model fits the experimental data notably well compared to alternative models. Utilizing regression analysis on the experimental ζ-potential data, the equation specific to the reduced cubic model is established and given in coded factors Eq. (7);

$$Zetapotential\ (mV) = -24.73-1.23X_1-0.1958X_2+1.37X_{3,1}+0.0725X_{3,0}-0.7886X_{3,-1}+0.1644X_1X_2-1.01X_1X_{3,1}+0.9454X_1X_{3,0}+0.7675X_1X_{3,-1}+0.7292\ X_2X_{3,1}-0.1725X_2X_{3,0}-0.4217X_2X_{3,-1}-1.22X_1^2-0.5341X_2^2+0.4931X_1X_2X_{3,1}-0.2969X_1X_2X_{3,0}-1.14X_1X_2X_{3,-1}-0.0401X_1^2X_{3,1}+2.37X_1^2X_{3,0}-1.55X_1^2X_{3,-1}+1.22X_2^2X_{3,1}-1.32X_2^2X_{3,0}-0.0899X_2^2X_{3,1} \quad (7)$$

In **Table S8**, the ANOVA results for the reduced cubic model reveal significant contributions from individual, second- and third-order, and interaction terms. The p-value (< 0.002) for the reduced cubic model confirms its substantial fit to the experimental data. Due to the sensitivity of ζ-potential values to various factors and their susceptibility to changes in temperature and duration, the experimental data are significantly dependent on these variables. For example, the fresh functionalized AuNP/aptamers with 100nm size were stable during first month. After 2 months the 100nm NPs were aggregated in comparison with the other sizes which were still stable after 2 months. Therefore, the correlation coefficient, with an R^2 value of 0.8126 for the reduced cubic model, ensuring a satisfactory fit by explaining a substantial proportion of the total variation in the predicted ζ-potential.

Table S8. *ANOVA for RSM variables fitted to Stability (ζ potential) Reduced Cubic model*

Source	Sum of Squares	df	Mean Square	F-value	p-value	Significance
**Model**	220.05	23	9.57	3.77	0.0020	significant
**X_1-AuNP Size**	36.43	1	36.43	14.36	0.0012	
**X_2-Aptamer Concentration**	0.9204	1	0.9204	0.3627	0.5538	
**X_3-Aptamer type**	90.76	3	30.25	11.92	0.0001	
X_1X_2	0.4323	1	0.4323	0.1704	0.6842	
X_1X_3	13.12	3	4.37	1.72	0.1945	
X_2X_3	17.42	3	5.81	2.29	0.1095	
X_1^2	15.03	1	15.03	5.92	0.0245	
X_2^2	2.89	1	2.89	1.14	0.2986	
$X_1X_2X_3$	10.12	3	3.37	1.33	0.2929	
$X_1^2X_3$	21.81	3	7.27	2.86	0.0624	
$X_2^2X_3$	8.30	3	2.77	1.09	0.3763	
**Residual**	50.75	20	2.54			
**Lack of Fit**	13.67	12	1.14	0.2458	0.9853	insignificant
**Pure Error**	37.08	8	4.64			
**Cor Total**	270.80	43				

Fitting of model and statistical analysis for different aptamers

For the RSM design experiments, a total of 44 runs were conducted to analyze the impact of four aptamer variables on various response variables, including ζ-potential (mV), surface coverage (pmol/cm^{-2}), bioconjugation efficiency (%), and yield (%), as per the FCCCD guidelines (**Table S4**). A face-centered design was employed to ensure that the operational range encompassed the area of interest. The p-values lower than 0.05 indicates that the developed model and the terms are statistically significant and values greater than 0.1 indicate the model terms are insignificant[5]. Values between 0.05 and 0.1 represent a zone of marginal or trending significance, suggesting potential, but not conclusive, statistical evidence. In this work, statistical significance was determined using a threshold of α = 0.05, with p-values lower than 0.05 indicating statistical significance.

Table S9. Summary of Model Fit Statistics for Key Response Variables

**Parameter**	Fitting model	R^2 value	P value	Supporting information

**Yield**	Quadratic model	0.9377	< 0.0001	Table S5
**Efficiency**	Reduced cubic model	0.9353	< 0.0001	Table S6
**surface coverage**	Quadratic model	0.9954	< 0.0001	Table S7
**ζ-potential**	Reduced cubic model	0.8126	0.002	Table S8

The validity of the proposed models was evaluated using the data presented in **Table S9**, which demonstrated that the model accurately represented each response. Additionally, the p-values were found to be lower than 0.0001 for surface coverage, bioconjugation efficiency, and yield, and 0.002 for ζ-potential, indicating the significance of the models. Furthermore, Lack of Fit tests showed no significant difference compared to pure error, affirming the accuracy of the model predictions. Detailed modeling results are provided in the supporting information.

Ligand and Particle concentrations

Table S10. *Nanoprobes; Ligand concentration, Particle concentration and number, and total surface area per mL for optimized bioconjugation experiments*

Nanoprobe	*Aptamer concentration (µM)*	*Number of particles per mL ($x10^{11}$)*	*Mass concentration (µg/mL)*	*Total surface area (m^2/mL) ($x10^4$)*
Au20nm-ApT2	*0.2 to 1*	*7.42*	*60*	*2.8*
Au50nm-ApT2	*0.2 to 1*	*0.47*	*60*	*0.176*
Au100nm-ApT2	*0.2 to 1*	*0.06*	*60*	*0.026*

Calibration lines for AuNPs

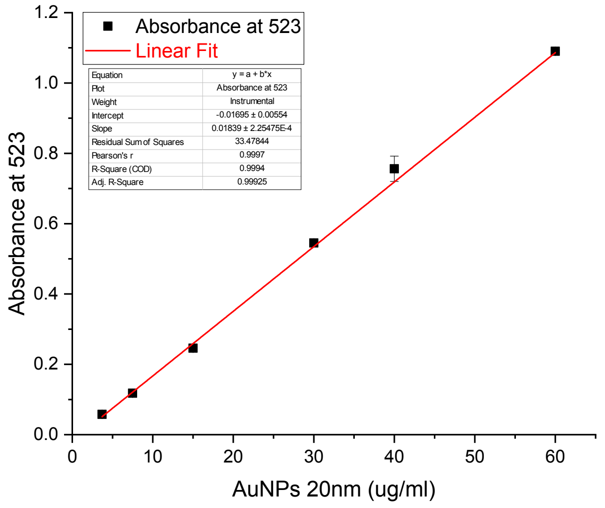

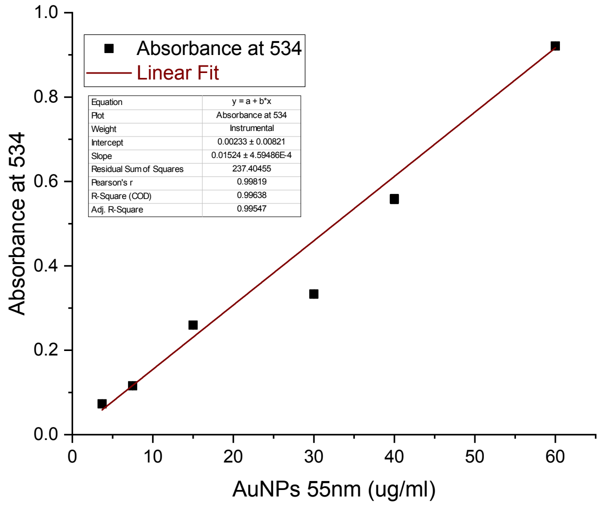

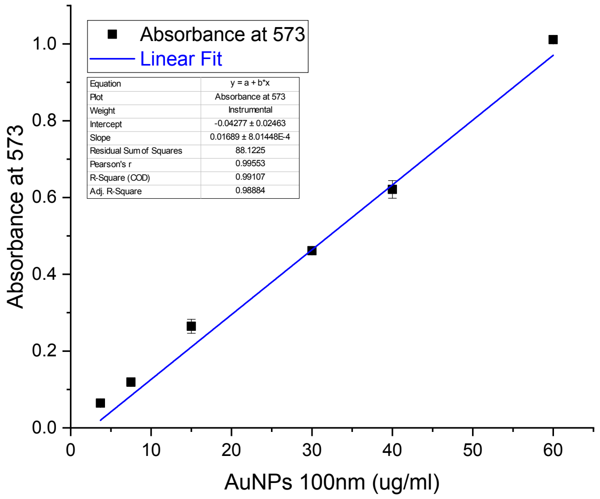

Figure S1. AuNPs calibration lines regarding the UV-vis absorbance at SPR peak

Calibration lines for aptamers

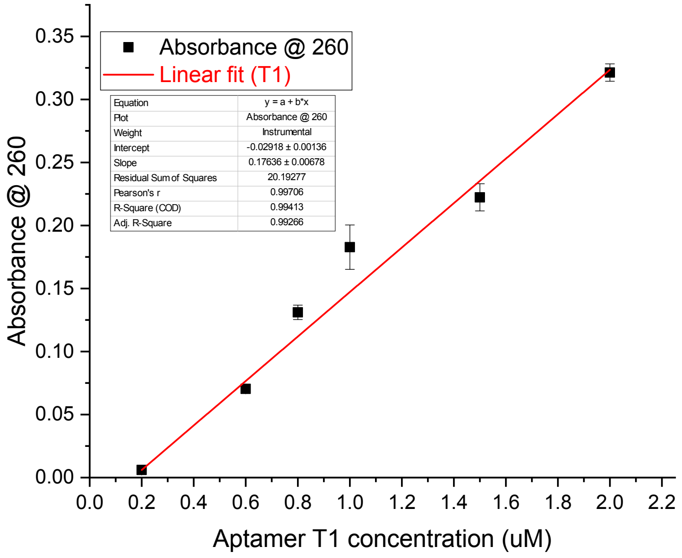

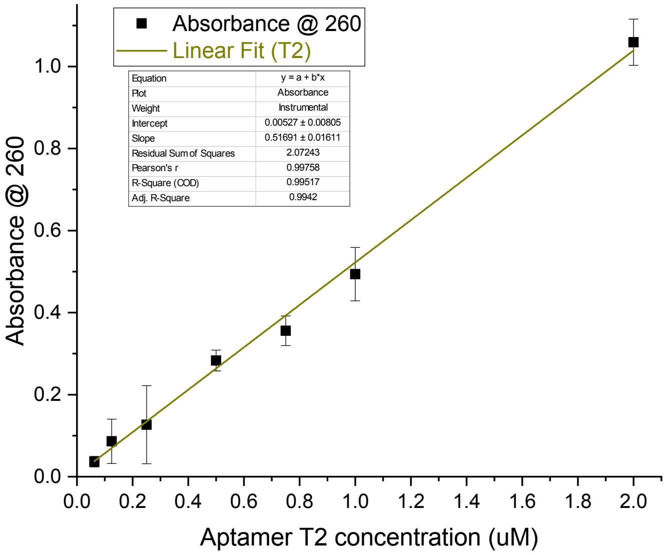

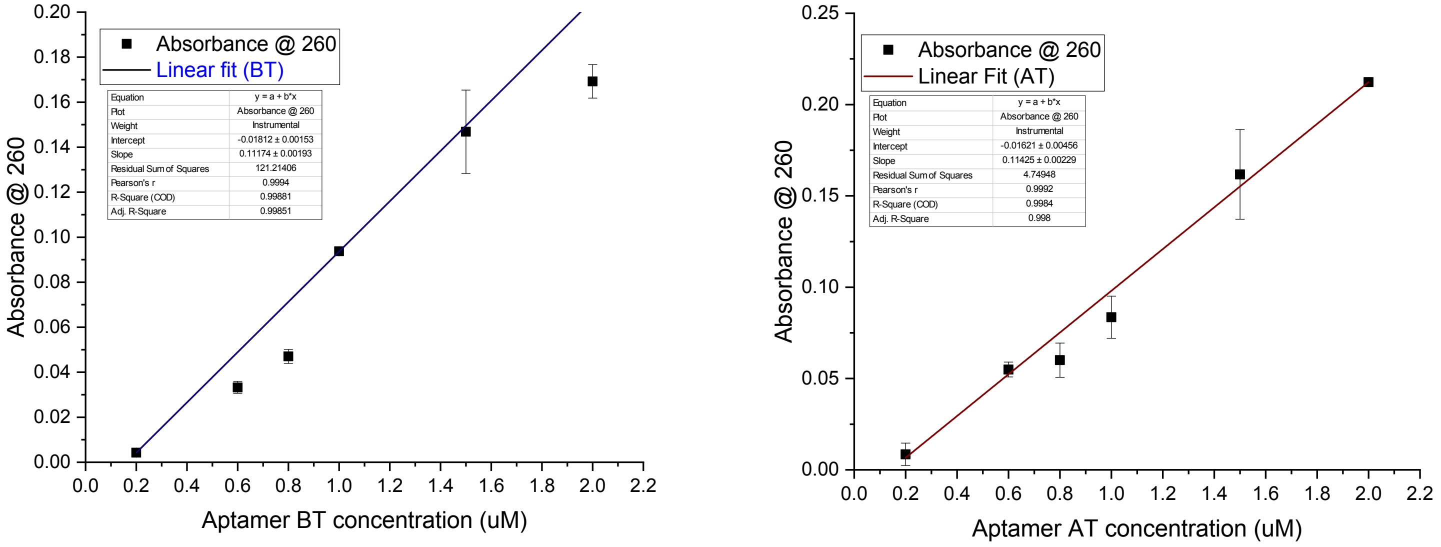

Figure S2. The calibration lines of different aptamers regarding the UV-vis absorbance at 260 nm

Optimization parameters

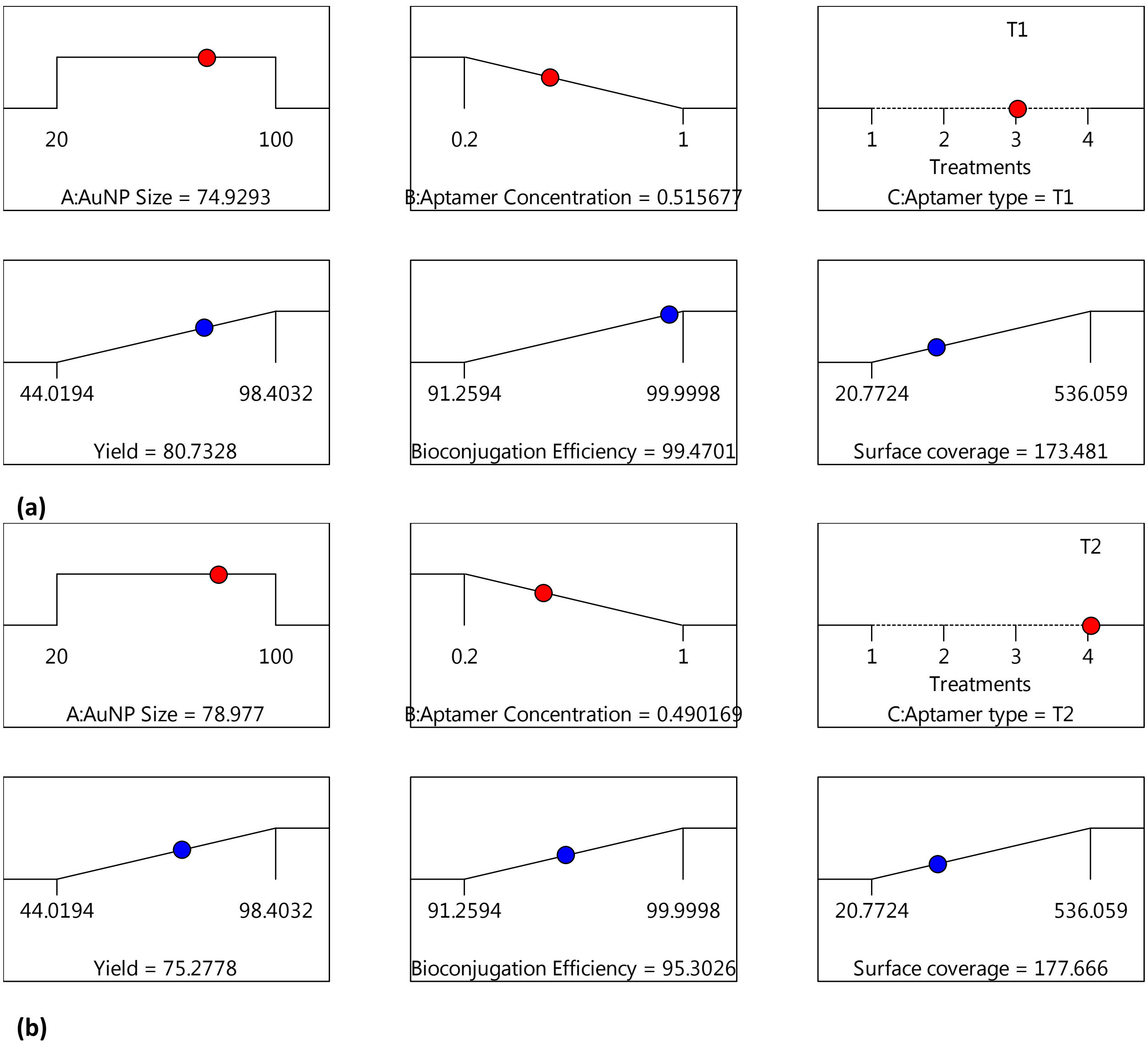

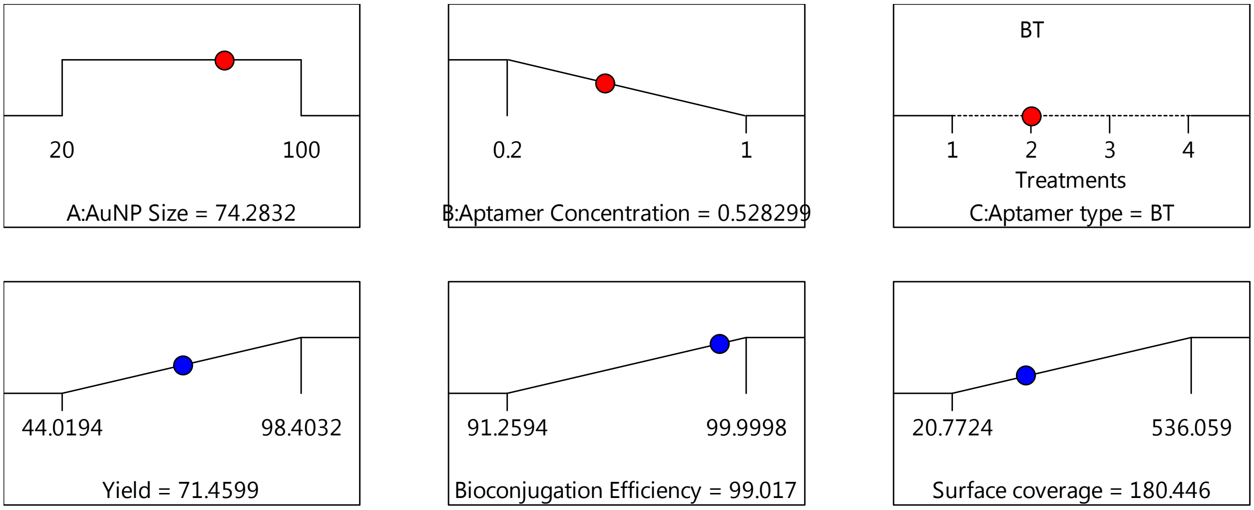

(c)

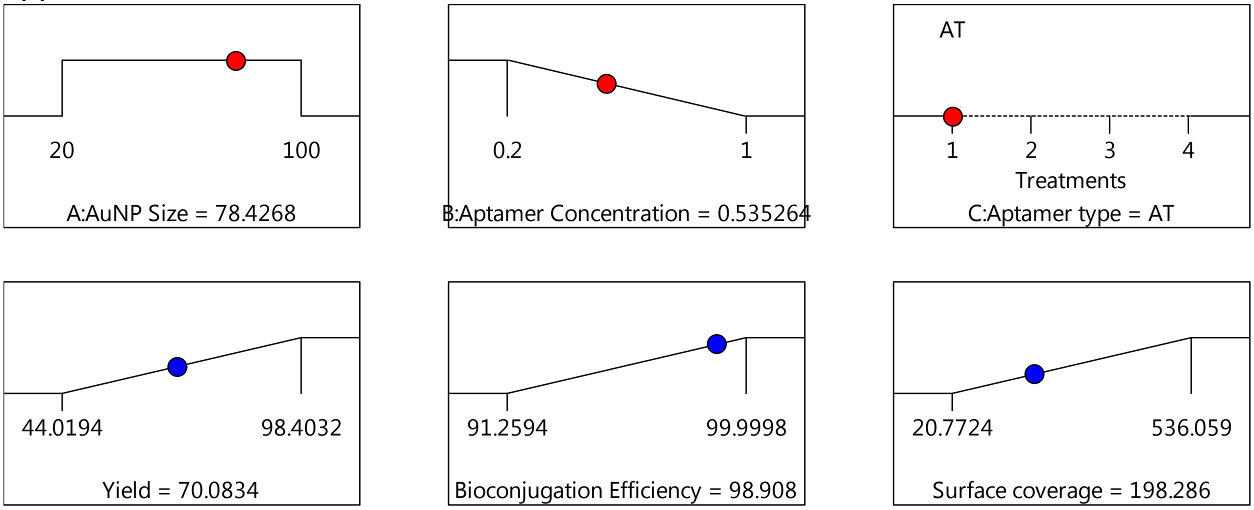

(d)

Figure S3. Numerical optimization parameter for a) aptamer T1, b) aptamer T2, c) aptamer BT, and d) aptamer AT

References

1. Zhang, X., Liu, S., Song, X., Wang, H., Wang, J., Wang, Y., Huang, J. and Yu, J. Robust and Universal SERS Sensing Platform for Multiplexed Detection of Alzheimer's Disease Core Biomarkers Using PAapt-AuNPs Conjugates. *ACS Sensors* **4**, 2140–2149 (2019).

2. Ziu, I., Laryea, E. T., Alashkar, F., Wu, C. G. & Martic, S. A dip-and-read optical aptasensor for detection of tau protein. *Anal. Bioanal. Chem.* **412**, 1193–1201 (2020).

3. Kim, S., Wark, A. W. & Lee, H. J. Femtomolar Detection of Tau Proteins in Undiluted Plasma Using Surface Plasmon Resonance. *Anal. Chem.* **88**, 7793–7799 (2016).

4. Bhattacharya, S. Central Composite Design for Response Surface Methodology and Its Application in Pharmacy. in *Response Surface Methodology in Engineering Science*

(IntechOpen, 2021). doi:10.5772/intechopen.95835.

5. Osman, M.J., Rashid, J.I.A., Khim, O.K., Yunus, W.M.Z.W., Noor, S.A.M., Kasim, N.A.M., Knight, V.F. and Chuang, T.C. Optimisation of a gold nanoparticle-based aptasensor integrated with image processing for the colorimetric detection of acephate using response surface methodology. *RSC Adv.* **11**, 25933–25942 (2021).
